# Supplementary material for: Ultrafast ion sieving using nanoporous polymeric membranes
Source: Nat Commun. 2018 Feb 8;9:569. doi: 10.1038/s41467-018-02941-6 (PMC5805712; doi:10.1038/s41467-018-02941-6)
Supplement: Supplementary file 1 — Supplementary Information [file 41467_2018_2941_MOESM1_ESM.pdf]

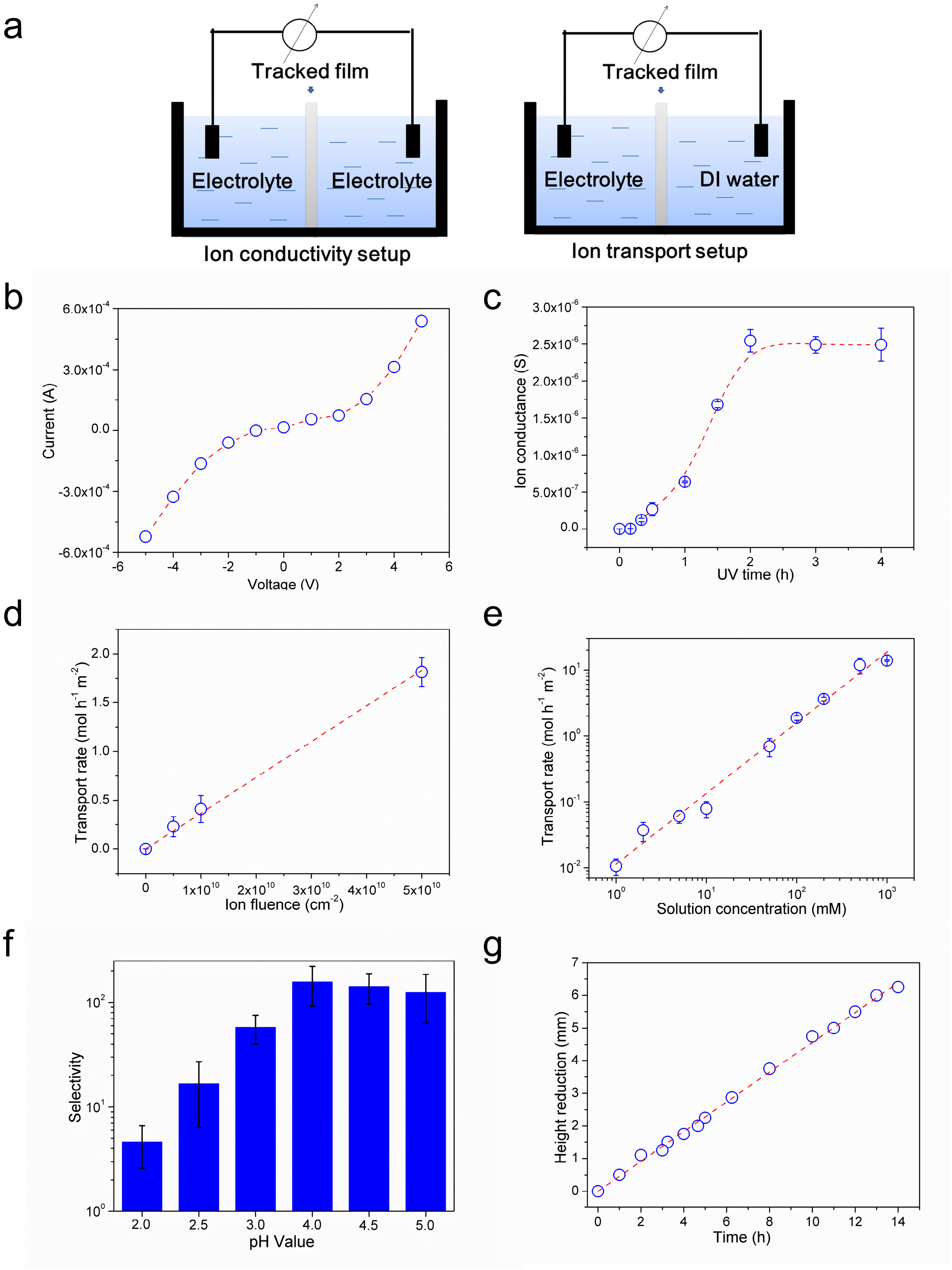

Supplementary Figure 1. Ionic transport properties of the PET Lumirror® membranes.

a. Scheme of the permeation measurement apparatus. For conductivity measurements, both the feed and permeate chambers are filled with electrolyte (left); for ionic transport measurements (ICP measurements), the permeate

chamber is filled with deionized (DI) water (right).

b. *I-V* curve of ionic conductance across the PET Lumirror® films (irradiated with 5 × 10^{10} Bi ions cm^{-2} and 4-hour UV radiation) measured with 0.1 M KCl solution in both chambers. No notable rectification effect was observed as the curve is nearly symmetric. It shows a typical activated shape, i.e., the slope increases as *V* increases, suggesting the transport is related to barriers such as the interaction between the ions and the pore wall or dehydration.

c. Ion conductance (circles) across the PET Lumirror® film (0.1 M KCl solution and applied voltage 1 V) as a function of UV exposure time. The dashed line is a guide for the eye.

d. Ionic transport rates (red squares) across the PET Lumirror® film (0.1 M KCl solution, 10 V applied voltage) as a function of irradiation fluence. For UV-treated samples, the conductance increases linearly over the entire range from 5×10^9 to 5×10^{10} ions cm^{-2}.

e. Ionic transport rates versus the KCl electrolyte concentration (applied voltage 10 V) following a power law relationship (exponent factor=1.07, R^2=0.986).

f. Selectivity of K^+/Mg^{2+} as a function of pH value. The transport rates of Mg^{2+} ions and K^+ ions through the PET Lumirror® films were measured with feed solutions of $MgCl_2$ (1 M) and KCl (1 M) at an applied voltage of 5 V. The films used in the measurements were irradiated with 1.4 GeV Bi ions at a fluence of 5×10^{10} ions cm^{-2} and subsequently exposed to UV radiation for 4 hours.

g. Evaporation rates of water during the transport measurements. The dashed line is a linear fit of the water height reduction (R^2=0.997) measured by means of a capillary inserted into the conductivity cell. The water evaporation rate, determined from the slope, is about 0.456 mm h^{-1}, which is corresponding to 25.33 mol h^{-1} m^{-2}.

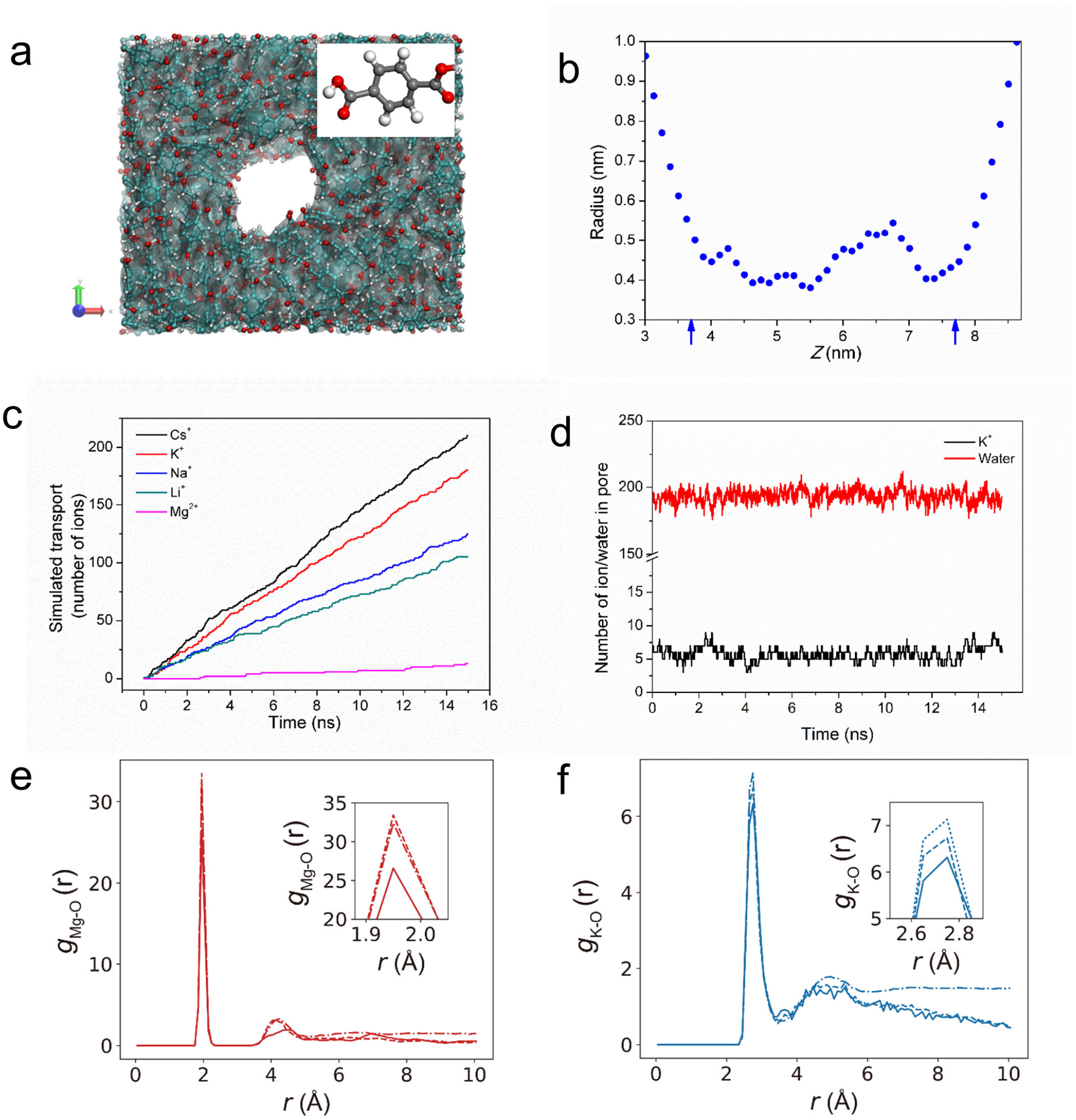

Supplementary Figure 2. Polymeric nanopore model and MD simulations.

a. Model of the polymeric nanopore. Carbon atoms, oxygen atoms and hydrogen atoms are represented as cyan, red and white. The insert shows the molecular structure of a protonated terminal of a PET chain. Carbon, oxygen and hydrogen atoms are colored as grey, red and white, respectively.

b. Distribution of the nanopore radius along the polymeric model nanopore. The radii were computed using the program HOLE[1]. The entrance and exit of the pore are at *z*=3.7 nm and 7.7 nm (arrows), respectively.

c. Transport fluxes of different simulated ions increase linearly with the simulation time.

d. Number of water molecules and K^+ ions is about 194 and 6, respectively， inside the nanopore.

e-f. Radial distribution function of water molecules surounding K^+ (**e**) and Mg^{2+} (**f**) ions in the bulk solution (dash line), charged nanopore region (solid line) and uncharged nanopore region (scatter). The inset shows the zoom in view of the first peak.

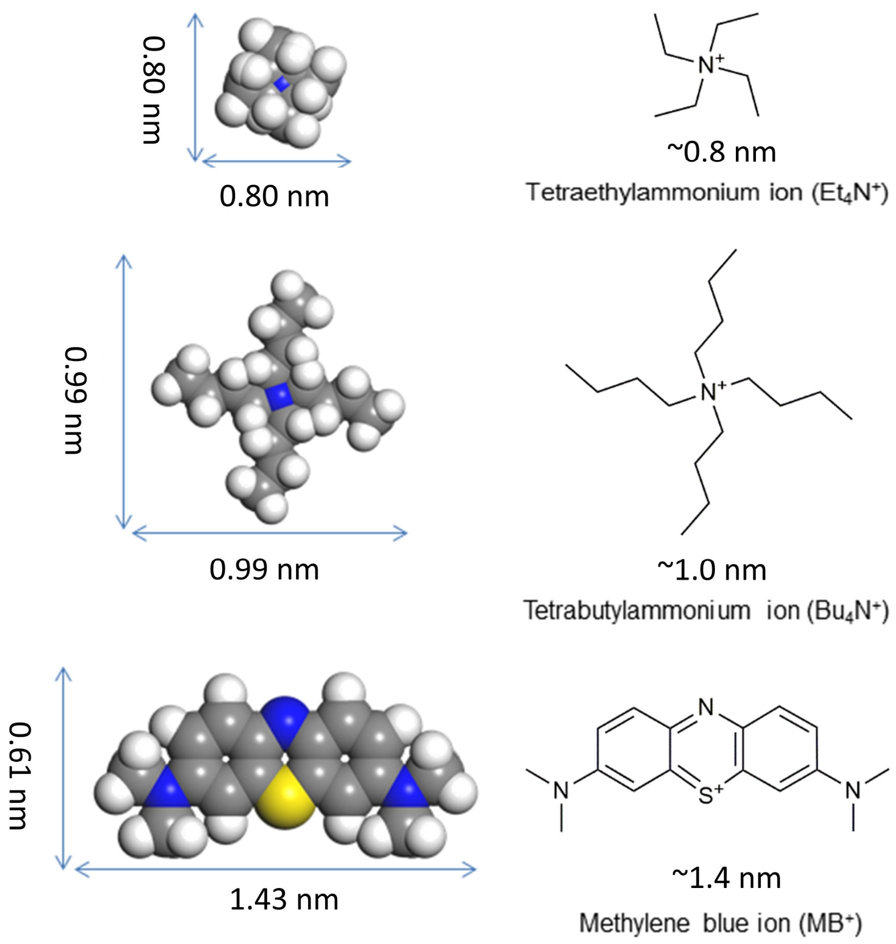

Supplementary Figure 3. Molecular structures of Bu_4N^+, Et_4N^+ and methylene blue ions.

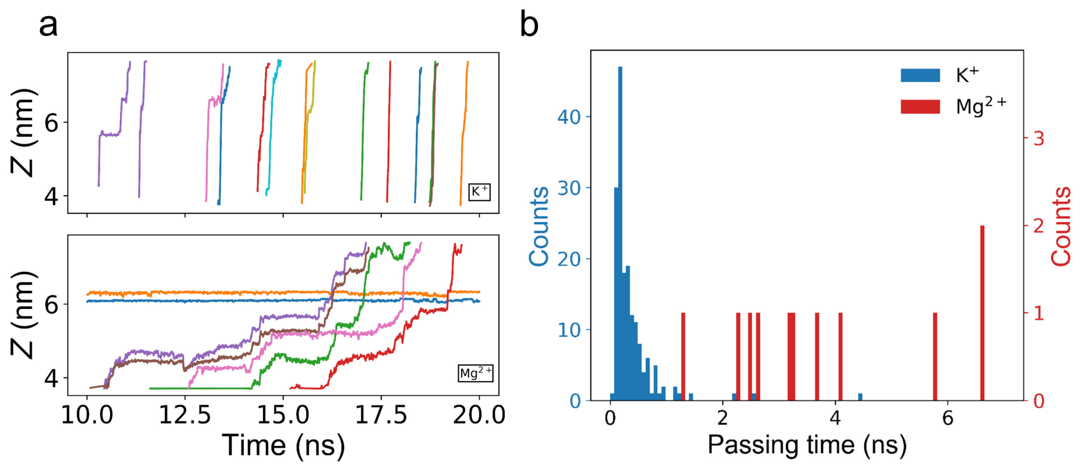

Supplementary Figure 4. MD simulations of cations transporting through the polymeric nanopore.

- **a.** Comparison of the typical trajectories of the transported K^+ (upper) and Mg^{2+} (lower) ions inside the nanopore.
- **b.** Histogram of the passing time of the K^+ (blue) and Mg^{2+} (red) ions transported through a nanopore.

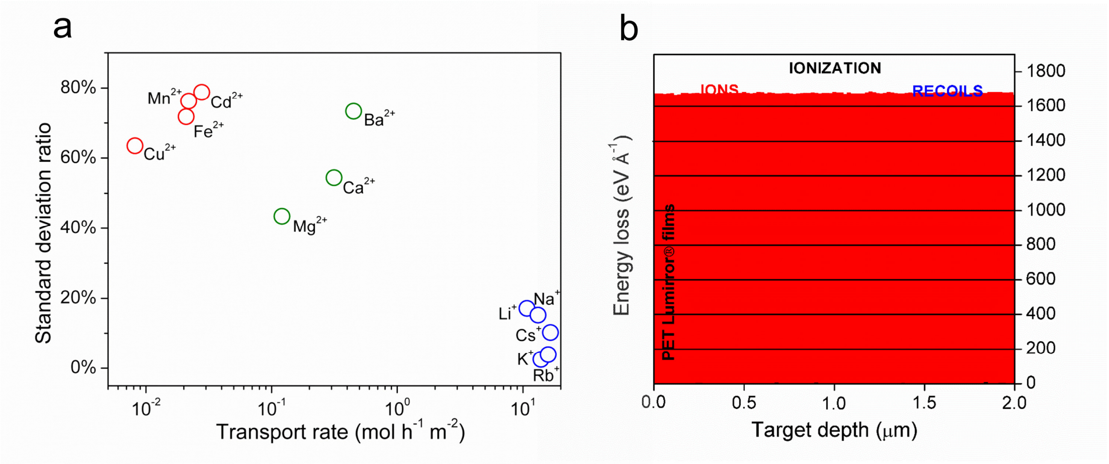

Supplementary Figure 5. Reproducibility of the transport measurements.

- **a.** The relative standard deviation of the transport rates (standard deviation/mean) of alkali metal ions (blue), alkaline earth metal ions (green), and transition metal and heavy metal ions (red) measured with ICP as a function of the mean transport rates. The transport rates are normalized per 1 M feed solution and measured under 10 V bias. The PET Lumirror® films used in the measurements were irradiated with 1.4 GeV Bi ions at a fluence of 5×10^{10} ions cm^{-2} and subsequently exposed to 4-hour UV radiation.
- **b.** Energy loss distribution in the 2-μm-thick PET Lumirror® films irradiated by 1.4 GeV Bi ions.

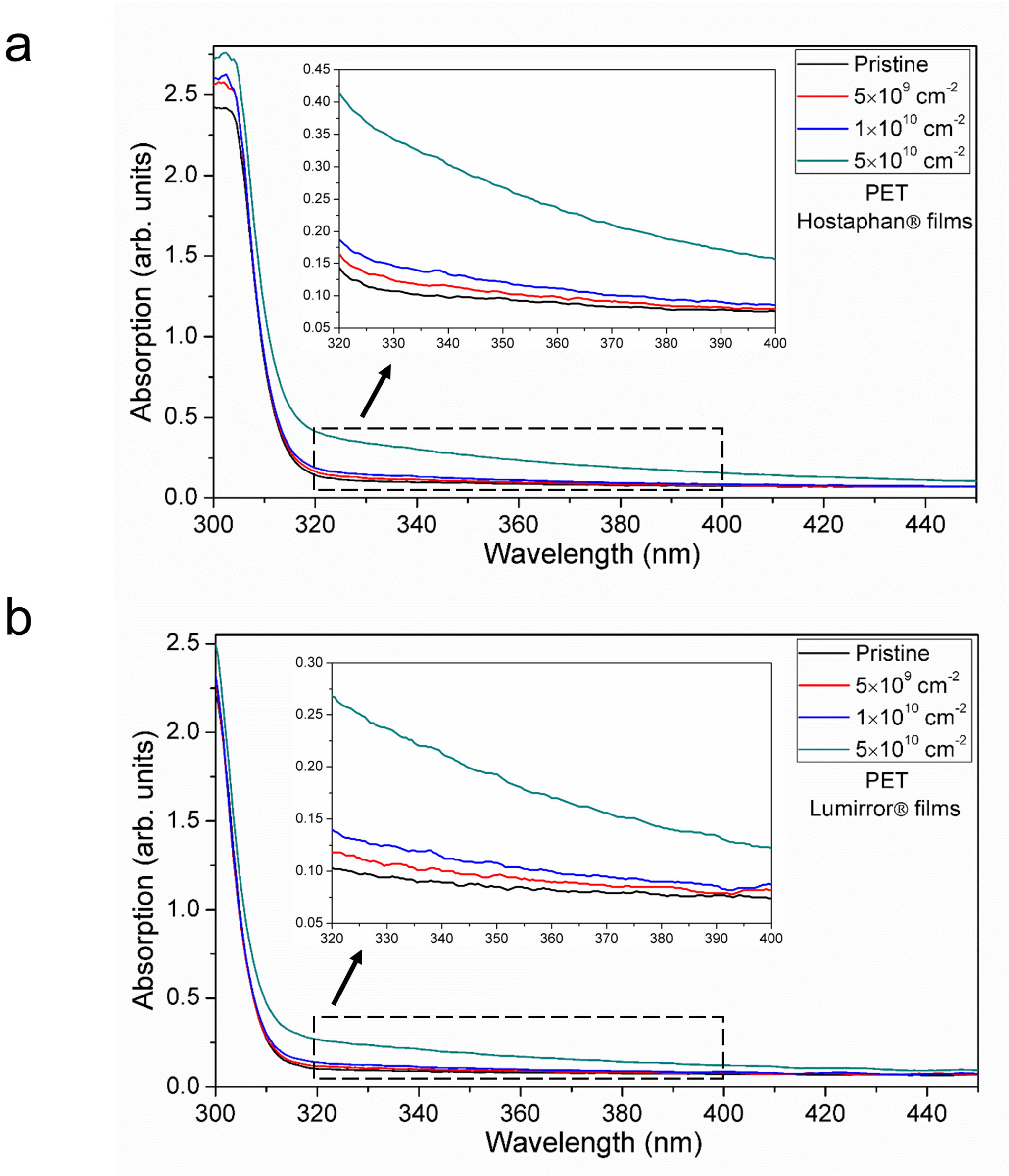

Supplementary Figure 6. UV-Visible absorption spectra of PET Lumirror® films (a) and PET Hostaphan® films (b) irradiated with different fluences.
Inserts are the zoom view of the wavelength rage of 320-400 nm.

Supplementary Table 1. List of analyzed ions and their experimental transport rates, naked radii, hydrated radii, and hydration energies.

Electrolyte species	Transport rate (mol h^{-1} m^{-2})	Standard deviation (mol h^{-1} m^{-2})	Naked radius[2] (Å)	Hydrated radius[2] (Å)	Hydration energy[3] (-kJ mol^{-1})
Li^+	10.8	1.80	0.6	3.82	475
Na^+	13.2	2.01	0.95	3.58	365
K^+	13.9	0.349	1.33	3.31	295
Rb^+	15.9	0.620	1.48	3.29	275
Cs^+	16.6	1.70	1.625	3.285	250
Mg^{2+}	0.121	0.0526	0.65	4.28	1830
Ca^{2+}	0.314	0.171	0.99	4.12	1505
Ba^{2+}	0.450	0.330	1.423	4.04	1250
Mn^{2+}	0.0219	0.0167	0.8	4.38	1760
Cd^{2+}	0.0278	0.0219	0.97	4.26	1755
Fe^{2+}	0.0209	0.0150	0.75	4.28	1840
Cu^{2+}	0.00819	0.00520	0.72	4.19	2010
Et_4N^+	1.403	0.512	3.48	4.00	/
Bu^4N^+	0.268	0.108	4.37	4.94	/
Methylene blue ion (MB^+)	3.812E-4	1.84E-4	/	7× 3	/
SO_4^{2-}	0.152	0.0597	2.3	3.79	1080
Cl^-	1.78	0.838	2.21	3.32	340
NO_3^-	1.59	0.472	1.29	3.35	300

Supplementary Table 2. Comparison of the maximum transport rates and ionic selectivities of different ionic transport membranes reported in literature.

Membrane	Max transport rate (K^+, Na^+, Li^+) (mol h^{-1} m^{-2})	Ionic selectivity (K^+/Mg^{2+}, Na^+/Mg^{2+}, Li^+/Mg^{2+})	Thickness (μm)	Driving force	Solution Concentration (M)
PET Lumirror® film in this study	13.9<br>13.2<br>10.8	134.58<br>113.17<br>90.00	2	Electric potential 10 V	1.00
[4]PET Hostaphan® film	0.00226<br>0.00346<br>0.0362	39.58<br>60.60<br>633.97	12	Electric potential 10 V	1.00
[5]NF-270	2.50<br>3.56<br>/	1.35<br>1.92<br>/	0.1	Pressure 2.758 MPa	0.0268<br>0.0342
[5]LLC (lyotropic liquid crystal)	1.26E-4<br>8.72E-5<br>/	32.78<br>22.75<br>/	40	Pressure 2.758 MPa	0.0268<br>0.0342
[5]SW30HR	0.00215<br>0.0108<br>/	1.11<br>5.55<br>/	0.1	Pressure 2.758 MPa	0.0268<br>0.0342
[6]Zwitterion Carbon Nanotube membrane (ZCNT)	11.9<br>12.2<br>/	~10.15<br>~10.41<br>/	~0.2	Pressure 0.100 MPa	0.0170
[7]Mxene	0.94<br>1.53<br>1.4	5.88<br>9.56<br>8.75	1.5	Pressure 0.495 MPa	0.2
[8]GO	~2.00<br>~2.00<br>/	1.00<br>1.00<br>/	5	Osmotic Pressure 2.476 MPa	1
[9]PSS threaded HKU ST-1-6.7 (PSS@HKU ST-1-6.7)	0.100<br>0.190<br>6.75	26.88<br>51.08<br>1815.00	8	Electric potential 0.4 V	0.5
[10]Graphene	/	/	0.0035	Osmotic pressure	0.0166

	1.14<br>/	7.70<br>/		0.079 MPa-Na^+,<br>0.059 MPa-Mg^{2+}	
[11]Physically confined GO (PCGO)	0.00650<br>0.00420<br>0.00500	650.00<br>420.00<br>500.00	1000	Osmotic pressure 2.476 MPa	1
[12]NF90	/<br>1.59E-3<br>/	/<br>300.00<br>/	~100	Osmotic pressure 2.800 MPa	1.15
[12]XLE	/<br>3.17E-4<br>/	/	~100	Osmotic pressure 2.800 MPa	1.15
[13]PET/P(AA-co-DEGMEM)	6.78<br>6.53<br>/	/	/	Osmotic pressure 0.248 MPa	0.1
[14]Sinergetic membrane	0.0600<br>0.0800<br>/	/	/	Osmotic pressure 0.619 MPa	0.25
[15]SLM	/<br>/<br>0.600	/	200	Pressure ~5.000 MPa	0.13
[16]Ion exchange membrane disulfonated poly(arylene ether sulfone)	/<br>0.0375<br>/	/	~100	Osmotic pressure 1.238 MPa	0.5

Supplementary Table 3. Binary ion selectivity of PET Lumirror® films& in 0.5 M XCl (X=K, Na, Li) and 0.5 M $MgCl_2$ solutions under the external bias voltage of 10 V.

Binary solution	Ion	Ionic transport rate (mol h^{-1} m^{-2})	Ion	Ionic transport rate (mol h^{-1} m^{-2})	Binary ion selectivity (X^+/Mg^{2+})
$KCl/MgCl_2$ solution	K^+	0.0342	Mg^{2+}	0.002050	16.70
$NaCl/MgCl_2$ solution	Na^+	0.0151	Mg^{2+}	0.000723	20.86
$LiCl/MgCl_2$ solution	Li^+	0.0143	Mg^{2+}	0.000673	21.21

& The films used in the measurements were irradiated with 1.4 GeV Bi ions at a fluence of 1×10^{10} ions cm^{-2} and subsequently exposed to 4-hour UV radiation.

Supplementary Table 4. Comparison of UV absorbance and absorption coefficients of PET Lumirror® films and PET Hostaphan® films irradiated with different ion fluences at the UV wavelength of 365 nm.

	PET Lumirror® films		PET Hostaphan® films	
Ion influence (cm^{-2})	Absorbance	Absorption coefficient (μm^{-1})	Absorbance	Absorption coefficient (μm^{-1})
0	0.085	0.03738	0.086	0.00738
5×10^{9}	0.090	0.04659	0.095	0.00911
1×10^{10}	0.096	0.05580	0.106	0.01122
5×10^{10}	0.163	0.13294	0.223	0.03370

Supplementary Table 5. Non-bonded interaction parameters used in the MD simulation.

Ion	$\sigma^{\&}$ (nm)	$\varepsilon^{\&}$ (kJ mol^{-1})	q (e)
Cs[17]	0.3741	0.79496	+1
K[17]	0.3142	0.36401	+1
Na[17]	0.2513	0.19623	+1
Li[18]	0.1800	0.07647	+0.75
Mg[17]	0.2111	0.06276	+2
Cl[17]	0.4044	0.62760	-1
Cl (Li)[18]	0.4417	0.49280	-0.75

& σ and ε are two key parameters to calculate the Lennard-Jones interaction, q is the charge of an ion.

Supplementary Note 1

Water transport and evaporation measurements through the nanoporous PET Lumirror® membranes.

The water transport experiments were performed using a sealed permeation measurement apparatus (Supplementary Fig. 1a). The 0.01 M KCl solution and deionized water were injected into the feed and permeate chamber, respectively. Then two capillaries (length=100 mm, inner radius r=0.5 mm, Huaxi Medical University Instrument Factory) were sealed in the two chambers separately. An external bias voltage of 10 V was applied between the two chambers, and the change of the liquid surface in the capillary was recorded as a function of time. As a control, the water evaporation rate was measured in the same way with no applied voltage. The measurements were conducted at room temperature.

For the water evaporation measurements, the height reduction of the water surface in the capillary evolved linearly over time. A linear fit yields $E = 0.456t$ (Supplementary Fig. 1g). The slope of this fit is the average evaporation rate of water, $R_e = 0.456$ mm h^{-1} = 25.33 mol h^{-1} m^{-2}. The amount of water evaporated (in mole) $n(E)$ is calculated with the following formula: $\rho(H_2O) \times E \times \pi \times r^2 = M(H_2O) \times n(E)$, where $\rho(H_2O)$ and $M(H_2O)$ is the density and molar mass of water, respectively.

For the water transport measurements, the amount of the water transported under an applied external voltage of 10 V was recorded in the same way. By subtracting the evaporation amount E from the total height reduction H, we obtained $n(T)$, the transport amount of water through the films, with the following equation: $\rho(H_2O) \times (H-E) \times \pi \times r^2 = M(H_2O) \times n(T)$. The amounts of transported ions were measured using ICP-atomic emission spectroscopy. Water as well as ions, both exhibit approximate linear relationships over time and the corresponding fits are: $n(H_2O) = 0.50t$, and $n(\text{ion}) = 0.08t$ (Fig. 2d). The slopes of the lines represent respective transport rates, i.e., the average amount of water and ions transported per square meter per hour

separately, or the water and ion transport rates. Thus, the average ratio of the transported water molecules over ions is $n(H_2O) / n(\text{ion}) = 6.3$.

Supplementary Note 2

UV absorption coefficient calculation

The absorbance was recorded in the UV-visible spectrum measurements. The absorption coefficient α is calculated with the formula[19]: $I/I_0 = (1-R)^2 e^{-\alpha L}$, where I_0 and I are the intensity of the incident and transmitted UV, respectively. R is the reflectivity of the incident light and L is the thickness of the film. R was calculated with the 'Fresnel formula' in the case of vertical light incidence: $R = (n_1-n_2)^2/(n_1+n_2)^2$, where n_1=1 is the refractive index of air and n_2=1.6 is the refractive index of PET Lumirror® film or PET Hostaphan® film according to their product characteristic parameters in their product manuals (Hoechst Hostaphan and Toray Industries Lumirror). This value is consistent with the measured value in the range of 1.54 and 1.65 based on the interference pattern observed in the UV-visible absorption spectrum. We confirmed that the calculated refractive indexes do not change as the film is irradiated with different ion fluence. This result indicates that the increase of the absorbance as the function of the irradiation fluence does not result from the change of reflection but the absorption.

Supplementary References

1. Smart, O. S., Neduvelil, J. G., Wang, X., Wallace, B. A. & Sansom, M. S. P. HOLE: A program for the analysis of the pore dimensions of ion channel structural models. *Journal of Molecular Graphics* **14**, 354–360 (1996).

2. E. R. Nightingale, 'Phenomenological Theory of Ion Solvation - Effective Radii of Hydrated Ions', *Journal of Physical Chemistry* **63**, 1381-87 (1959).

3. Marcus Y. A simple empirical model describing the thermodynamics of hydration of ions of widely varying charges, sizes, and shapes [J]. *Biophysical Chemistry* **51**, 111-127 (1994).

4. Wen Q, Yan D, Liu F, et al. Highly Selective Ionic Transport through Subnanometer Pores in Polymer Films[J]. *Advanced Functional Materials* **26**, 5796-5803 (2016).

5. Hatakeyama E S, Gabriel C J, Wiesenauer B R, et al. Water filtration performance of a lyotropic liquid crystal polymer membrane with uniform, sub-1-nm pores [J]. *Journal of membrane science* **366**, 62-72 (2011).

6. Liu T Y, Yuan H G, Li Q, et al. Ion-responsive channels of zwitterion-carbon nanotube membrane for rapid water permeation and ultrahigh mono-/multivalent ion selectivity[J]. *ACS nano* **9**, 7488-7496 (2015).

7. Ren C E, Hatzell K B, Alhabeb M, et al. Charge-and size-selective ion sieving through Ti3C2T x MXene membranes[J]. *The journal of physical chemistry letters* **6**, 4026-4031 (2015).

8. Joshi R K, Carbone P, Wang F C, et al. Precise and ultrafast molecular sieving through graphene oxide membranes[J]. *Science* **343**, 752-754 (2014).

9. Guo Y, Ying Y, Mao Y, et al. Polystyrene Sulfonate Threaded through a Metal–Organic Framework Membrane for Fast and Selective Lithium ‐ Ion Separation [J]. *Angewandte Chemie* **28**, 15344-15348(2016).

10. O'Hern S C, Jang D, Bose S, et al. Nanofiltration across defect-sealed nanoporous monolayer graphene[J]. *Nano letters* **15**, 3254-3260 (2015).

11. Abraham J, Vasu K S, Williams C D, et al. Tunable sieving of ions using graphene oxide membranes[J]. *Nature Nanotechnology* **12**, 546-550 (2017).

12. Somrani A, Hamzaoui A H, Pontie M. Study on lithium separation from salt lake brines by nanofiltration (NF) and low pressure reverse osmosis (LPRO) [J].

Desalination **317**, 184-192 (2013).

13. Smolinska K, Bryjak M, Wolska J, et al. pH-sensitive membranes for lithium separation [J]. *Materials Chemistry and Physics* **148**, 548-553 (2014).

14. Garifzyanov A R, Davletshina N V, Garipova A R, et al. Sinergetic membrane extraction of lithium ions with new organophosphorus carriers[J]. *Russian Journal of General Chemistry* **84**, 285-288 (2014).

15. Song J, Li X M, Zhang Y, et al. Hydrophilic nanoporous ion-exchange membranes as a stabilizing barrier for liquid–liquid membrane extraction of lithium ions[J]. *Journal of Membrane Science* **471**, 372-380 (2014).

16. Cassady H J, Cimino E C, Kumar M, et al. Specific ion effects on the permselectivity of sulfonated poly (ether sulfone) cation exchange membranes [J]. *Journal of Membrane Science* **508**, 146-152 (2016).

17. Brooks, B. R. et al. CHARMM: The Biomolecular Simulation Program. *Journal of Computational Chemistry* **30**, 1545–1614 (2009).

18. Pluhařová, E., Mason, P. E. & Jungwirth, P. Ion Pairing in Aqueous Lithium Salt Solutions with Monovalent and Divalent Counter-Anions. *Journal of Physical Chemistry A* **117**, 11766–11773 (2013).

19. Hecht E. Optics 4th edition [J]. Optics, 4th Edition, Addison Wesley Longman Inc, 1998.
