# Supplementary material for: Ultrafast ion sieving using nanoporous polymeric membranes
Source: Nat Commun. 2018 Feb 8;9:569. doi: 10.1038/s41467-018-02941-6 (PMC5805712; doi:10.1038/s41467-018-02941-6)
Supplement: Supplementary file 3 — Description of Additional Supplementary Files [file 41467_2018_2941_MOESM3_ESM.pdf]

Description of Additional Supplementary Files

File Name: Supplementary Movie 1
Description: A 3 ns simulation of K^+ ions passing through a polymer pore. The polymer is shown as transparent material; negatively charged residues (COO^-) are shown in red (O) and cyan (C); K^+ ions are shown in pink. Cl^- and water are not shown. During the simulation, a voltage bias of 0.3 V nm^{-1} is applied along the pore axis.

File Name: Supplementary Movie 2
Description: A 3 ns simulation of Mg^{2+} ions passing through a polymer pore. The polymer is shown as transparent material; negatively charged residues (COO^-) are shown in red (O) and cyan (C); Mg^{2+} ions are shown in blue. Cl^- and water are not shown. During the simulation, a voltage bias of 0.3 V nm^{-1} is applied along the pore axis.
